# Methods for Estimating Human Joint and Endpoint Stiffness

**DOI:** 10.3390/s26217052

**Published:** 2026-11-07

**Authors:** Salvatore Maria Li Gioi, Francesca Cordella, Loredana Zollo

**Affiliations:** Research Unit of Advanced Robotics and Human-Centred Technologies, Università Campus Bio-Medico di Roma, 00128 Rome, Italy

**Keywords:** biomechanics, joint stiffness, endpoint stiffness, mechanical impedance, system identification, EMG-driven modeling, musculoskeletal modeling, machine learning, rehabilitation robotics

## Abstract

Mechanical stiffness is the displacement-dependent component of mechanical impedance. In joint coordinates, it describes the local mapping from small angular displacements to changes in joint torque; when using Cartesian coordinates, endpoint stiffness describes the corresponding mapping from small spatial displacements to changes in endpoint force. Both quantities are relevant to neuromechanical assessment and to applications in rehabilitation and physical human–robot interaction. However, selecting an estimation method remains difficult because stiffness is not estimated and validated consistently across studies. This review provides an application-oriented synthesis of methods for estimating human joint and endpoint stiffness. Following study inclusion, methods were classified along a mutually exclusive primary axis as either perturbation-response identification, in which stiffness is derived from the mechanical response to a deliberately imposed perturbation used for identification, or non-perturbative inference, in which stiffness is inferred from measurements acquired without such an identification perturbation. Experimental conditions and sensing or modeling choices were retained as orthogonal descriptors rather than additional methodological classes. Because standardized head-to-head comparisons against common reference measurements remain scarce, the available evidence does not support a universal ranking of estimation accuracy. The proposed benchmarking framework and decision tree, therefore, match method selection to application requirements rather than prescribing a globally superior approach. The synthesis further shows that reproducible use requires a precise definition of what is estimated and under which conditions, together with sufficient methodological detail to replicate the estimation and validation procedures.

## 1. Introduction

Human movement depends not only on the forces generated by the neuromusculoskeletal system, but also on its ability to regulate how the limb mechanically responds to external disturbances. Mechanical stiffness captures the local change in this response when the limb is displaced from its current state. Its modulation allows the limb to remain compliant during unconstrained motion, become stable during physical interaction and resist unexpected perturbations. Stiffness estimation can, therefore, reveal aspects of neuromuscular function that cannot be inferred from kinematics or force alone. However, stiffness is not directly observable: its interpretation depends on the coordinate space in which it is expressed, the biomechanical state in which it is evaluated and the procedure used to estimate it. These elements should be defined rigorously before the available methods can be meaningfully compared.

Stiffness represents the displacement-dependent component of mechanical impedance and should not be used as a synonym for the complete impedance response, which also includes inertia- and damping-related contributions. At a specified operating state and under static conditions, joint and endpoint stiffness are locally defined as

(1) $$K_{q}=\frac{\partial \tau }{\partial q},\;K_{x}=\frac{\partial F}{\partial x},$$

where τ and F denote the external joint torque and endpoint force required to produce small changes in joint configuration q and endpoint position x, respectively. For a single degree of freedom, these definitions correspond locally to the ratio between a small change in torque or force and the associated angular or spatial displacement. In multidimensional systems, $K_{q}$ and $K_{x}$ are matrices whose off-diagonal elements also describe mechanical coupling across coordinates [1,2,3].

These definitions apply locally at a specified operating state and should be distinguished from quasi-stiffness, which is the slope of a torque–angle trajectory during voluntary movement. If activation, velocity, reflex state or external loading changes along the trajectory, its slope need not represent local mechanical stiffness. The two may coincide for small excursions around one operating point when relevant state variables remain effectively constant and inertial, damping and reflex effects are negligible or have been accounted for.

A related distinction concerns coordinate space: joint and endpoint stiffness are related but non-interchangeable quantities and their local relationship depends on the limb Jacobian J(q). Consequently, this relationship varies with joint configuration. The same joint stiffness may, therefore, produce different endpoint responses when posture changes, while an endpoint estimate cannot generally be assigned to a specific joint without additional measurements or modeling assumptions [4,5].

Muscle stiffness represents a distinct level of mechanical description. It characterizes the local slope of the muscle force–length relationship and contributes to joint stiffness through musculoskeletal geometry and muscle moment arms [6]. Joint stiffness also incorporates the mechanical effects of passive tissues and, according to the activation state and observation interval, intrinsic and reflex-mediated muscle responses. Muscle, joint and endpoint stiffness should, therefore, not be treated as equivalent quantities. This review focuses on stiffness estimated at the joint or endpoint level. Studies reporting only isolated muscle or tendon stiffness are outside its scope unless those quantities are propagated to one of these two coordinate spaces.

The coordinate space alone does not fully determine the meaning of a stiffness estimate. The estimate also depends on the neuromechanical state of the participant and on the time scale over which the response is observed. Passive stiffness is assessed while voluntary activation is minimized and is intended primarily to characterize the mechanical contribution of passive structures. Residual muscle activation and reflexes elicited by the experimental protocol may, nevertheless, affect the measured response. Active stiffness is assessed during voluntary force production or movement and includes the activation-dependent intrinsic response of the neuromuscular system. Reflex-mediated contributions may also be captured when the duration and bandwidth of the observation allow them to develop. During movement, the operating state continuously changes; stiffness must, therefore, be interpreted as a local and potentially time-varying property rather than as an invariant characteristic of the limb.

This dependence on neuromechanical state is precisely what makes stiffness informative. In motor-control research, joint- and endpoint-stiffness estimation provides a means of investigating how the nervous system stabilizes the limb and adapts its response to changing interactions [7,8,9]. The same mechanical information can support clinical assessment by revealing changes in passive resistance or neuromuscular coordination associated with injury and neurological impairment [10,11,12]. When physical interaction is mediated by an assistive or robotic device, an estimate of the user’s stiffness may be used either to adapt the assistance to the user’s current behavior [13,14] or to endow robotic devices, such as prostheses, with more bio-inspired mechanical behavior [15,16,17].

Because stiffness cannot be observed in isolation, it must be estimated from measurements that contain information about its mechanical effects. Perturbation-response identification obtains this information by deliberately introducing a mechanical input and analyzing the resulting deviation from the operating state or ongoing trajectory. In human stiffness-estimation scenarios, a perturbation is defined as a controlled and quantifiable mechanical excitation, such as an applied force, torque or displacement, that produces a measurable deviation from the limb’s operating state or ongoing trajectory. Voluntary motion alone is, therefore, not considered a perturbation unless a deliberately elicited and quantified transient serves as the identification input. This approach establishes a mechanical input–output relationship, but requires sufficiently informative excitation and appropriate compensation for the dynamics of the measurement apparatus. Moreover, the identification input may alter the neuromuscular state under investigation by disrupting the task or eliciting reflex responses.

Non-perturbative inference estimates stiffness without applying an identification perturbation during use. Instead, it relies on a calibrated computational relationship between stiffness and physiological or mechanical measurements. Its ability to operate without interrupting the task makes this approach particularly relevant to continuous monitoring and robotic control. Its validity, however, depends on whether the underlying model is identifiable and whether the estimator remains reliable outside the conditions used for calibration or training. The distinction between perturbation-response identification and non-perturbative inference, therefore, concerns the source of the information used to obtain the estimate. It does not depend on the sensing technology, since physiological signals may complement mechanical measurements in either approach.

The suitability of an estimation method consequently depends on the intended use rather than on accuracy alone. Controlled clinical assessment requires estimates that can be interpreted and reproduced across sessions or participants. Continuous robotic control introduces different constraints because the estimate must be available with sufficiently low latency and remain robust without identification inputs that interfere with the task. Evidence cannot be transferred uncritically between these contexts, particularly when the studies concern different anatomical regions, coordinate spaces or operating states. Variation in experimental excitation, observation bandwidth, model assumptions and validation procedures further restricts direct comparison. The current literature, therefore, does not support a universal ranking of stiffness-estimation methods.

Although previous works have examined the integration of stiffness-estimation formulations [18] and compared specific identification methods [19], a broader synthesis linking estimation principles, validation evidence and application requirements is still needed.

Therefore, this review addresses this need through a systematic and application-oriented synthesis of methods for estimating human joint and endpoint stiffness. Estimation methods were organized along a mutually exclusive primary axis according to whether stiffness was obtained through perturbation-response identification or non-perturbative inference. Biomechanical conditions, sensing technologies and computational implementations were retained as orthogonal descriptors rather than treated as competing methodological classes. Within this framework, the review examines what each approach estimates, the assumptions that support its interpretation and the evidence provided for its validation. The resulting benchmarking framework and decision tree connect method selection with application requirements, with the aim of clarifying when an estimate is valid rather than prescribing a universally superior estimator.

Therefore, the manuscript is organized as follows: Section 2 describes the review design, search strategy, eligibility criteria, screening workflow and data-extraction fields; and Section 3 presents the proposed taxonomy of human joint stiffness-estimation methods and introduces the main methodological categories considered in this review. The following sections synthesize the reviewed studies: Section 4 provides a critical comparison of the methods, discusses their validation evidence and application constraints and introduces the flowchart for application-oriented method selection; and Section 5 summarizes the main findings, current limitations and future research directions.

## 2. Literature Selection and Scope of the Review

This paper was prepared as a review aimed at providing a structured and critical overview of human joint and endpoint stiffness-estimation methods. This topic was selected because the reviewed literature is highly heterogeneous. Therefore, the objective was not to estimate a pooled effect size or to rank methods according to a single outcomes, but rather to organize the field, compare methodological assumptions and identify current limitations and future research needs.

### 2.1. Literature Search

The literature search was conducted using Scopus, IEEE Xplore and PubMed, with the latest update performed on 20 October 2026 to include the term "Endpoint" in the search strategy. The following Boolean search string was applied across all three databases: ("Joint" OR "Endpoint") AND ("Stiffness" OR "Impedance") AND ("Estimation" OR "Assessment").

In each database, the search was restricted to the title, abstract and keyword fields. In Scopus, the results were further refined using the keyword “Biomechanics” because the search retrieved a large number of records that were not relevant to the scope of this review. No publication-date restrictions were applied; therefore, each database was searched across its entire available coverage up to the date of the final search. The results were restricted to English-language journal articles.

All records retrieved from the three databases were imported into Zotero. Potential duplicates were identified using the Zotero Duplicate Items function and manually verified before the corresponding records were merged. Following deduplication, title and abstract screening and subsequent full-text assessment were performed independently by the first and second authors according to the predefined eligibility criteria. Disagreements were resolved through discussion, with the other co-author consulted when consensus could not be reached. No additional relevant review articles were identified.

To complement the database search and minimize the risk of overlooking relevant studies, backward- and forward-citation searching was performed for all included studies. Specifically, backward-citation searching involved manually examining the reference lists of the included articles to identify potentially relevant publications, whereas forward-citation searching involved identifying subsequent publications citing these studies through Scopus, IEEE Xplore and PubMed. The complete study-selection process, including records identified through the initial database search and subsequent updates, duplicate removal, screening, eligibility assessment and final inclusion, is reported in Figure 1. Backward- and forward-citation searching did not identify additional eligible studies and, therefore, did not affect the reported counts.

### 2.2. Study Selection

Studies were included if they investigated living human participants or human-derived data; estimated joint-level or endpoint stiffness of an upper or lower limb; reported a quantitative stiffness value or quantitative validation outcome; and were full-length, peer-reviewed journal articles in English. Studies were excluded if they reported only isolated muscle or tendon stiffness without propagation to joint or endpoint coordinates, estimated damping or inertia without a stiffness term, contained no quantitative result, did not concern human biomechanics or were abstracts, editorials, theses, patents or non-peer-reviewed reports.

The database searches yielded 3314 records, of which 1400 were duplicates. Of the 1914 unique records screened, 1846 were excluded at the title and abstract screening stage, leaving 68 articles for full-text assessment. Fourteen articles were excluded because they reported no quantitative stiffness data (six), addressed isolated muscle stiffness without propagation to joint or endpoint coordinates (five), or estimated damping without a stiffness term (three). The final synthesis, therefore, included 54 distinct studies, which are individually reported in Table 1, Table 2, Table 3 and Table 4: nine passive perturbation-response studies, 11 active static or quasi-static perturbation-response studies, 13 dynamic perturbation-response studies, and 21 non-perturbative inference studies.

### 2.3. Methodological Synthesis and Appraisal

Table 1, Table 2, Table 3 and Table 4 summarize each study by estimation method; anatomical target and degrees of freedom; participant number and population where reported; model; validation approach; and reported outcomes.

After inclusion, the full methods sections were coded to develop the taxonomy. The primary classification question was whether stiffness was identified from the measured response to an imposed or experimentally elicited mechanical transient. Studies satisfying this condition were assigned to perturbation-response identification; all others were assigned to non-perturbative inference. Each study was then described using secondary axes: joint versus endpoint coordinate space, passive versus active and static versus dynamic task state, sensing modality, actuator type and computational model. Thus, EMG can be an auxiliary measurement in either primary class and does not determine eligibility or class membership.

Because joints, coordinate spaces, tasks, inputs, models and validation metrics were not commensurate, outcomes were not pooled. Method classes were appraised according to validation strength, identifiability, computational and calibration burden, real-time feasibility, sensitivity to subject and sensor variability, reproducibility and application constraints. These dimensions support a structured comparison but not a universal accuracy ranking.

## 3. Stiffness-Estimation Methods’ Taxonomy

Due to the large number of stiffness-estimation methods proposed in the literature, the first objective of this work was to categorize and organize them in a taxonomy, with the aim of guiding the readers to select the most suitable strategy for their specific study.

Perturbations may be externally imposed by a clinician, robot or orthosis, or be experimentally elicited as a transient within the task. Voluntary movement alone is not labeled a perturbation unless its mechanical transient is explicitly used as the identification input. Therefore, the taxonomy in Figure 2 divides stiffness-estimation methods according to whether an identification perturbation is applied. *Perturbation-response identification* estimates stiffness from the mechanical response to an identification input. It includes passive, active-static and active-dynamic conditions. *Non-perturbative inference* estimates stiffness without an identification perturbation during intended operation. It includes data-driven mappings and neuromusculoskeletal model-based inference, including reduced Hill-type models and full simulation environments.

Coordinate space, task state, sensing technology and computation are secondary descriptors. A study may estimate joint or endpoint stiffness, may be passive or active and static or dynamic and may use force/torque sensors, kinematics, EMG, electrical impedance tomography or other physiological signals [20].

For the descriptive distributions, each study was assigned once to a primary methodological principle, one dominant implementation technology and one primary stiffness coordinate space. Studies estimating joint stiffness were then assigned to one primary anatomical joint. For hybrid studies, the dominant implementation technology was defined as the component directly responsible for the final stiffness estimate rather than auxiliary sensing, calibration or validation procedures. Figure 3 reports the descriptive distribution of the 54 studies retained in the revised classification. Perturbation-response identification accounts for 33 studies (61.1%), whereas 21 studies (38.9%) use non-perturbative inference. Among the 54 studies, the dominant implementation is robotic or instrumented in 29, reduced musculoskeletal modeling in nine, data-driven or EMG mapping in seven, simulation-engine modeling in five, clinician/manual assessment in three and simulation-only identification in one. The primary stiffness coordinate space is the joint in 42 studies and the endpoint in 12. Among the joint-space studies, the primary anatomical targets are ankle (11), elbow (9), shoulder (7), knee (7), wrist (6) and hip (2).

### 3.1. Perturbation-Response-Identification Methods for Passive Stiffness

Passive protocols seek to minimize voluntary activation while a manual or instrumented input displaces the limb. Manual assessment is accessible and clinically familiar but its input waveform depends on the examiner. Robotic and orthotic devices improve input repeatability and synchronized sensing, although their attachment and dynamics must be included in the measurement model [12,21,22,23,24].

Among the passive-stiffness studies summarized in Table 1, ref. [10] assessed ankle stiffness through instrumented stretching in individuals with multiple sclerosis, while [11] compared elbow stiffness between paretic and contralateral limbs following stroke. These studies demonstrate the potential of passive stiffness measurements to characterize neuromuscular impairments, although such comparisons do not establish absolute accuracy against an independent stiffness reference.

**Table 1:** Summary of passive perturbation-response-identification approaches. D–P, F/E, RUD and DoF denote dorsiflexion–plantarflexion, flexion–extension, radio-ulnar deviation and degree(s) of freedom, respectively. *N* denotes participant number; a number without a population descriptor refers to able-bodied subjects. K-ref. indicates direct comparison of estimated stiffness with an independent reference or known value (✓, reported; and ×, not reported). NR indicates that the corresponding information was not reported or that the validation approach could not be identified.

Ref.	Perturb.	Joints	DoF	N	Model	Validation	K Ref.	Outcomes
[12]	Robotic exoskeleton	Upper limb	3 joint axes	11 post-stroke, 12 able-bodied	$\Delta \tau =K_{q}\Delta \theta$	Post-stroke group comparison	×	Stiffness/coupling increased post-stroke
[21]	Wrist robot	Wrist	2: F/E, RUD; 1: PS	10 (EMG follow-up: 5; repeatability subset: 4)	Stiffness ellipse	Torque–angle model fit; test–retest (1 month)	×	Fit $R^{2}$: 0.958/0.928/0.605; repeatability: 9%
[22]	Manual translation	Shoulder	1 translation	15 able-bodied, 16 with shoulder impairments	$F=K_{x}\Delta x$	Clinical device assessment	×	NR
[23]	Pendulum test	Knee	1: F/E	15 with paraplegia (SCI), 15 able-bodied	Viscoelastic pendulum	Group comparison	×	Group difference p=0.01
[24]	Ankle orthosis	Ankle	1: D–P	9	$\Delta \tau =K_{q}\Delta \theta$	Optical tracking comparison; test–retest	×	Repeatability: ICC >0.846, SEM <1.38
[25]	Pendulum test	Knee	1: F/E	96	Damped pendulum	Model fitting; repeated-measure reliability	×	Repeatability: moderate–high ICC
[26]	Wrist robot	Wrist	2: F/E, RUD	10	Subject-specific model	Torque–angle regression; subject-specific vs. simplified model	×	Fit $R^{2}$ (F/E, RUD): 0.73±0.13/0.90±0.07
[10]	Instrumented rotation	Ankle	1: D–P	13 with spastic MS, 10 able-bodied	Intrinsic/reflex response	Patients vs. controls	×	Stiffness change: +138% flexors, +152% extensors
[11]	Passive displacement	Elbow	1: F/E	10 with hemiparetic stroke, 10 able-bodied controls	Joint torque–angle	Paretic vs. contralateral	×	Side difference: not significant

In [22], the authors applied shoulder translation manually, measured humeral displacement with magnetic tracking and measured force with a load cell. The reported quantity is translational shoulder stiffness given in force–displacement coordinates,

(2) $$F=K_{x}\Delta x,$$

where *F* is applied force, Δx is humeral translation and $K_{x}$ is the local slope. Instead, in [23] the authors used a clinician-administered Wartenberg pendulum test and a viscoelastic knee model [25]. After estimating the natural frequency, rotational stiffness was obtained as

(3) $$\begin{array}{c} \omega_{d}=\frac{2\pi }{T},\;\omega_{n}=\frac{\omega_{d}}{\sqrt{1-\zeta^{2}}}=\sqrt{\frac{K^{\prime }}{J}},\\ K_{q}=K^{\prime }-\frac{mgl}{2} \end{array}$$

where *J* is the segment moment of inertia about the knee axis, *T* is the observed oscillation period, ζ is the damping ratio and *m*, *l*, and *g* are the anthropometric and gravitational parameters. The formulation assumes a linearized, underdamped pendulum, with $\omega_{n}\approx \omega_{d}$ only when damping is negligible.

End-effector measurements directly identify stiffness at the contact coordinate. Recovering the stiffness of one or more anatomical joints additionally requires sufficient joint kinematics, force/torque information and an identifiable mapping. The most distal joint is not recovered automatically. Formica et al. [21] used a dedicated three-degree-of-freedom wrist robot together with the MIT-MANUS platform [27] to characterize wrist flexion–extension, radio-ulnar deviation and their coupling (Figure 4), comparing ellipse fitting and multiple regression approaches. Similarly, Giovannetti et al. [26] employed a subject-specific wrist kinematic model. For ankle stiffness assessment, Zhang et al. [24] estimated dorsiflexion–plantarflexion stiffness using the quasi-static torque–angle relationship.

Formica et al. compared ellipse fitting and multiple regression, whereas [24] estimated ankle dorsiflexion–plantarflexion stiffness from

(4) $$\Delta \tau =K_{q}\Delta \theta ,$$

where Δτ and Δθ are the torque and angular deviations. Their quasi-static reduction assumes negligible orthosis friction and negligible acceleration- and velocity-dependent torques. These assumptions are valid only when the transmission is direct-drive or friction-compensated and the mobilization is sufficiently slow and approximately constant-speed.

An exoskeleton can instrument several joint axes directly. Zhang et al. [12] estimated coupled shoulder, elbow and wrist stiffness during slow passive mobilization (Figure 5).

A simplified Newton–Euler model [28] related the vector of torque deviations to angular deviations,

(5) $$\left[\begin{array}{c} \Delta \tau_{1}\\ \Delta \tau_{2}\\ \Delta \tau_{3} \end{array}\right]=\left[\begin{array}{ccc} K_{11} & K_{12} & K_{13}\\ K_{21} & K_{22} & K_{23}\\ K_{31} & K_{32} & K_{33} \end{array}\right]\left[\begin{array}{c} \Delta \theta_{1}\\ \Delta \theta_{2}\\ \Delta \theta_{3} \end{array}\right]$$

where indices 1–3 denote the instrumented shoulder, elbow and wrist axes. Diagonal terms describe same-joint stiffness, whereas off-diagonal terms describe cross-joint coupling. Slow constant-speed mobilization was used to reduce inertia and damping contributions.

At class level, passive perturbation-response methods offer a mechanically interpretable local slope. Instrumented systems provide superior control of waveform and synchronization; manual protocols offer portability and clinical accessibility. Neither is an absolute ground truth: manual estimates are operator-dependent, while instrumented estimates depend on alignment, attachment, device compensation and model order.

### 3.2. Perturbation-Response Identification of Active Stiffness

Active protocols maintain voluntary muscle activation while stiffness is identified. In a static or quasi-static condition, the nominal joint configuration is held while small perturbations are applied; in a dynamic condition, stiffness is allowed to vary along a movement and must be identified within a time-local window. EMG may quantify activation or co-contraction in either condition, but it is an auxiliary signal and does not change the primary perturbation-response classification.

Lee et al. [29] used an impulsive 23-kgf pulley load to produce a coupled two-degree-of-freedom response comprising sagittal-plane shoulder extension and elbow flexion. Endpoint force at the hand was recorded with a force transducer and shoulder–elbow kinematics with an Optitrak system (Figure 6).

For this particular pulley implementation, inference depends on synchronization among independent force and motion systems and on the compliance, backlash and release dynamics of the cable–weight transmission. Joint-specific mechatronic platforms reduce these sources of uncertainty by collocating actuation and sensing [30,31]. Kuxhaus et al. [30] aligned the elbow rotation axis with a stiffness tester, fixed the slightly pronated forearm and measured motor torque, angle and acceleration. Under slow constant-speed perturbation, they fitted the quasi-static relation

(6) $$\tau \left(t\right)=K_{q}\theta \left(t\right)+\tau_{0},$$

where $\tau_{0}$ is a torque offset. The omission of inertia and damping is specific to that quasi-static operating range and is not an inherent property of mechatronic platforms.

End-effector robots can apply multi-directional perturbations, but they identify endpoint stiffness directly; joint stiffness is obtained only when the multi-joint mapping is observable. Perreault et al. [32] used a two-joint planar manipulandum [33] with the arm held in a fixed, subject-specific horizontal-plane shoulder–elbow configuration. Equal-magnitude endpoint forces were directed along the elbow–hand line (S, requiring predominantly shoulder torque), along the hand–shoulder line (E, predominantly elbow torque) and midway between them (SE, requiring both torques). Planar stochastic position perturbations and a multiple-input/multiple-output recursive estimator quantified how force direction constrained the voluntary orientation of static endpoint stiffness (Figure 7).

Related quasi-static studies omitted inertia and damping only after restricting the perturbation bandwidth and analysis window [4,34]. A three-dimensional robot is not required merely because shoulder and elbow stiffness are estimated together: planar instrumentation is sufficient for planar flexion–extension models. Spatial actuation becomes necessary when the target includes out-of-plane degrees of freedom or a full three-dimensional endpoint stiffness matrix. Fang et al. [35] used a seven-degree-of-freedom KUKA LWR 4+ to combine endpoint force/displacement, upper-limb kinematics and an activation-dependent model for multi-joint stiffness (Figure 8),

(7) $$K_{q}=J_{M}^{T}\left(q\right)a_{cc}K_{s}J_{M}\left(q\right)$$

where $J_{M}\left(q\right)$ maps joint-angle changes to muscle-length changes, $K_{q}$ is the joint stiffness matrix and $K_{s}$ contains muscle-stiffness coefficients. The dimensionless scalar $a_{cc}$ is a co-contraction index derived from normalized biceps and triceps EMG, rather than an unspecified “activity index”. The main features of passive and active static or quasi-static perturbation-response studies are summarized in Table 2.

**Figure 8:**
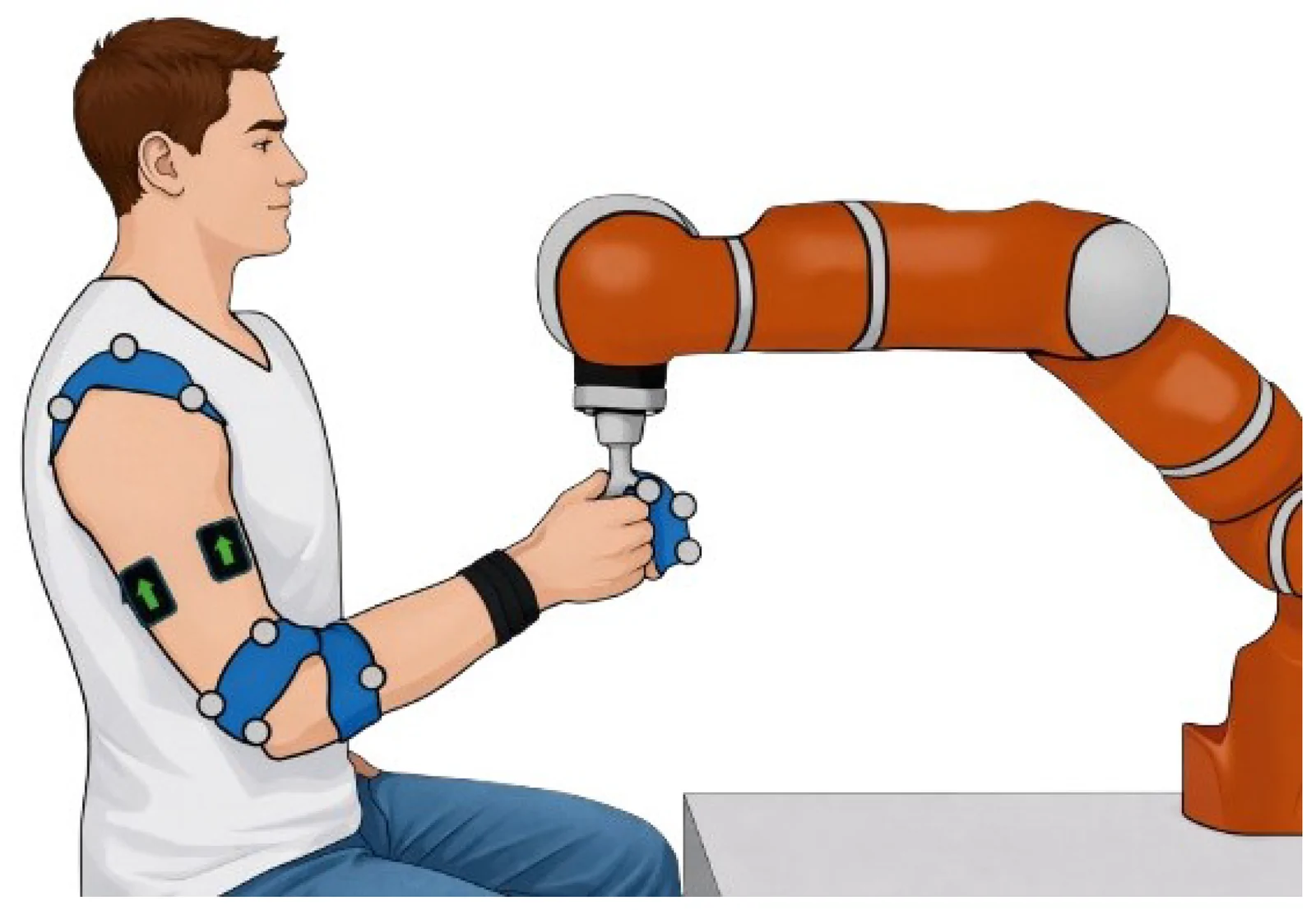
Example of the experimental setup used in [35].

**Table 2:** Summary of active static or quasi-static perturbation-response-identification approaches. D–P, F/E, RUD and DoF denote dorsiflexion–plantarflexion, flexion–extension, radio-ulnar deviation and degree(s) of freedom, respectively. *N* denotes participant number; a number without a population descriptor refers to able-bodied subjects. K-ref. indicates direct comparison of estimated stiffness with an independent reference or known value (✓, reported; and ×, not reported). NR indicates that the corresponding information was not reported or that the validation approach could not be identified. Outcomes refer to the corresponding validation procedure and are not directly comparable measures of stiffness accuracy.

Ref.	Perturb.	Joints	DoF	N	Model	Validation	K Ref.	Outcomes
[29]	Pulley system	Shoulder, Elbow	2: shoulder extension, elbow flexion	38	Four-link manipulator	Torque reconstruction (model fit)	×	Model-fit NR
[35]	KUKA LWR IV	Upper limb	All	2	Equation (7)	Separate test trials vs. perturbation-derived reference	✓	Error vs. perturbation reference: 19.8%, 21.6%
[4]	Robot end-effector	Endpoint	2-D endpoint	10	$F=K_{x}\Delta x$	Mechanical-spring benchmark	✓	Spring benchmark error <5%
[34]	Robot end-effector	Endpoint	2-D endpoint	24	$F=K_{x}\Delta x$	Restoring-force fit: symmetric stiffness component	×	Fit $R^{2}=0.87$ (range 0.58–0.97)
[32]	Robot end-effector	Endpoint	2-D endpoint	4	$\begin{array}{c} I\ddot{x}+B\dot{x}\\ +K_{x}x=F \end{array}$	Online–offline estimator comparison	×	Online/offline differences: orientation 0.91±5.85°; shape 3±6%; area 12±15%
[36]	Ankle orthosis	Ankle	1: D–P	8	$K_{q}=A/B$ (least squares)	Gait-control assessment	×	Stiffness accuracy NR
[30]	Mechatronic platform	Elbow	1: F/E	10	Equation (6)	Comparison with/without damping	×	Damping effect on *K*: −0.30 to +0.13 Nm/rad (8/480 records)
[31]	Mechatronic platform	Ankle	1: D–P	10	$\begin{array}{c} \tau =I\ddot{\theta }+B\dot{\theta }\\ +K_{q}\theta \end{array}$	Torque reconstruction (model fit)	×	Torque VAF: 98±1.2%
[5]	Robot end-effector	Endpoint	2-D endpoint	10	Second-order impedance	Force/posture comparison	×	Stiffness NR
[37]	Robot-assisted tooling	Wrist	1: RUD	1	$\tau =B\dot{\theta }+K_{q}\theta +\tau_{0}$	Robot-stiffness comparison	✓	Robot-reference difference: 2.8%
[38]	Ramp-and-hold	Wrist	1: F/E	10	$\Delta \tau =K_{q}\Delta \theta$	Repeated post-exercise tests	×	Post-exercise *K*: 0.299→0.254 Nm/deg

Under dynamic conditions, stiffness is time-varying because joint configuration, muscle length, contraction velocity, activation and reflex state evolve during the task. The estimator must, therefore, separate inertia, damping and stiffness within a finite window while preserving sufficient temporal resolution. The neuromuscular response is time-varying rather than “constant” and its stabilizing role can be stated technically as reducing trajectory-tracking error and rejecting disturbances. Identification inputs have been delivered by mechatronic platforms [39,40], robots or exoskeletons [33,41,42,43,44,45] and controlled task-release transients [9].

In [9], twenty participants generated a prescribed endpoint force against a WAM robot and, after an unpredictable hold interval, the handle constraint was released. Force and displacement during the ballistic transient were fitted with a second-order endpoint model using continuous-time instrumental-variable identification. The study is informative because EMG co-activation was recorded, yet stiffness class membership is determined by the release response used for mechanical identification. The estimated endpoint stiffness was task-dependent and approximately invariant across tested movement directions, illustrating predictive tuning at different arm configurations.

Liao et al. [42] constrained the arm to a simplified three-dimensional triangular geometry and used a Franka Emika Panda robot to estimate dynamic endpoint stiffness. The constraint reduces the configuration space; it does not make stiffness independent of configuration. Configuration and anthropometry remain embedded in the mapping matrix V and $D_{s}$,

(8) $$K_{x}=V\;A_{cc}D_{s}V^{T}$$

where $K_{x}$ is endpoint stiffness, $A_{cc}$ represents activation-dependent scaling and $D_{s}$ represents anthropometric scaling along the principal axes.

Gomi et al. [43] modeled the upper limb as a planar shoulder–elbow linkage. This reduces the number of degrees of freedom but does not remove configuration dependence. A linearized joint-space perturbation model was written as

(9) $$I\delta \ddot{q}+\frac{\partial H}{\partial \dot{q}}\delta \dot{q}+\left(\frac{\partial I\ddot{q}}{\partial q}+\frac{\partial H}{\partial q}\right)\delta q=-D\delta \dot{q}-R\delta q+\delta \tau_{\mathrm{ext}}$$

where I and H denote the inertia matrix and Coriolis–centrifugal vector, D and R denote joint damping and stiffness and $\tau_{ext}$ is external torque. Once R is identified, endpoint stiffness can be obtained as

(10) $$K_{x}={\left(J^{T}\right)}^{-1}\left(R+\frac{\partial J^{T}}{\partial q}F_{\mathrm{arm}}\right)J^{-1},$$

where J is the configuration-dependent Jacobian, assumed to be square and nonsingular, and $F_{\mathrm{arm}}$ is the endpoint force generated by the arm, which is opposite to the externally applied force. The term involving the Jacobian derivative and the endpoint force accounts for load-dependent geometric stiffness. The mapping is valid locally away from singular configurations. Equation (10) makes explicit that posture matters even in a two-degree-of-freedom system.

Lee and Hogan [45] estimated two-degree-of-freedom ankle impedance from pre-swing through early stance using Anklebot perturbations [46] (Figure 9).

In the case of 13 healthy participants walking on a treadmill at their preferred speed, random torque perturbations were applied using the Anklebot for approximately 13 min, yielding more than 550 gait-cycle realizations. Ankle impedance was identified from pre-swing through swing to early stance. The identified impulse-response functions (IRFs) predicted the perturbation-induced ankle-angle output with VAFs of 87.6% in inversion–eversion (I–E) and 89.7% in dorsiflexion–plantarflexion (D–P). When the IRFs were approximated by second-order inertia–viscosity–stiffness models, the corresponding angle-output VAFs were 83.6% and 85.7%, respectively. The second-order model fit to the IRF exceeded 90% across subgait phases, except during pre-swing in D–P (85.6%).

Transfer-function and impulse-response methods have also been evaluated through simulations and experiments [19,47,48,49]; simulation-only validation tests algorithmic recovery under known parameters but does not establish robustness to human or device uncertainty.

At class level, active dynamic perturbation-response identification provides the closest mechanical input–output characterization during movement, but its estimates depend on excitation richness, window length, model order and compensation of time-varying robot and limb dynamics. Reporting should include model fit together with bias, variance, temporal tracking, repeatability and the operating trajectories over which parameters are identifiable. Table 3 summarizes the main implementations.

**Table 3:** Summary of perturbation-response-identification approaches under dynamic or time-varying conditions. D–P, I–E, F/E, IRF, DoF and VAF denote dorsiflexion–plantarflexion, inversion–eversion, flexion–extension, impulse response function, degree(s) of freedom and variance accounted for, respectively. *N* denotes participant number; a number without a population descriptor refers to able-bodied subjects. K-ref. indicates direct comparison of estimated stiffness with an independent reference or known value (✓, reported; and ×, not reported). NR indicates that the corresponding information was not reported or that the validation approach could not be identified. Outcomes refer to different validation procedures and are not directly comparable measures of stiffness accuracy.

Ref.	Perturb.	Joints	DoF	N	Model	Validation	K Ref.	Outcomes
[41]	LOPER interface	Hip; ankle	1: F/E, D–P	2	$\tau =I\ddot{\theta }+B\dot{\theta }+K_{q}\theta$	Simulation and torque reconstruction	✓	Stiffness error: 3.5–11.5%
[45]	Exoskeleton	Ankle	2: I–E, D–P	13	IRF identification; second-order approximation	Second-order model fit to IRF	×	IRF fit: >90%; pre-swing D–P: 85.6%; stiffness accuracy NR
[42]	Robot end-effector	Endpoint	3-D endpoint	NR	$K_{x}=VA_{cc}D_{s}V^{T}$	Model calibration and testing	×	Model error: 10.51%
[44]	Robot end-effector	Endpoint	3-D endpoint	9	Stiffness ellipsoid	Posture and task comparison	×	Stiffness accuracy NR
[9]	Controlled handle release	Endpoint	1-D per direction	19	Second-order model; instrumental variables	Model fitting	×	Model fit: >88%
[43]	Robot manipulandum	Shoulder, elbow	2: planar	3	Joint–endpoint mapping (Equation (10))	Torque reconstruction	×	Torque-fit $R^{2}$: 0.799–0.806
[47]	Multiaxis perturbations	Endpoint	Multi-axis	NR	IRF	Simulation and dynamic-response identification	×	NR
[19]	Simulated and robotic perturbations	Ankle	1: D–P	1	Time-varying identification methods	Simulation and experimental fit	✓	Experimental VAF: 74.8–87.2%
[48]	Simulated and imposed motion	Ankle	1: D–P	5	State-space model	Simulation and experiments	×	NR
[49]	Simulated perturbations	Elbow	1: F/E	N/A	Non-parametric identification	Simulation benchmark	×	NR
[39]	Mechatronic platform	Ankle	1: D–P	5	PV-IRF; intrinsic/reflex model	Simulation and torque prediction	✓	Torque-prediction VAF: 92.8–93.6%
[40]	Mechatronic platform	Elbow	1: F/E	5	$\tau =I\ddot{\theta }+B\dot{\theta }+K_{q}\theta$	Movement reconstruction	×	NR
[33]	Robot manipulandum	Endpoint	2-D endpoint	NR	MIMO dynamic IRF	Known physical-system benchmark	×	NR

### 3.3. Experimental Determinants Shared Across Perturbation-Response Methods

Whether estimating passive or active stiffness, the result is local to the point of operation and is valid only if the protocol ensures that the parameters can be identified. Reports should, therefore, specify the joint configuration, the load on the end effector, the activation target and the amplitude of the perturbation. In multi-jointed limbs, the stiffness of the joints and the end effector remains dependent on posture via the Jacobian, even in planar models with two degrees of freedom. Planar devices are sufficient for shoulder and elbow flexion–extension under planar conditions, while spatial robots are necessary only for out-of-plane degrees of freedom or for the three-dimensional estimation of end-effector stiffness. Unless adequately compensated for, the apparatus is part of the measured input–output system. Therefore, if this is not the case, the reports should describe the limb–device coupling and any mechanical, control or acquisition dynamics that may influence the estimation. Friction can be neglected only in direct-drive systems or in those with demonstrable compensation, while inertial and damping effects can be omitted only under sufficiently slow and quasi-static conditions with negligible residual dynamics. Perturbations should exceed measurement noise without leaving the local linear range. Their bandwidth and the observation window should also make clear whether the estimate includes only the intrinsic response or captures reflex-mediated and voluntary contributions.

### 3.4. Non-Perturbative Inference of Stiffness

Non-perturbative methods infer stiffness from physiological, kinematic or kinetic signals without applying an identification perturbation during intended use. This makes them candidates for continuous estimation in prosthetic, exoskeleton and tele-impedance control, but the output is conditional on the calibrated mapping or biomechanical model rather than being identified directly from an online mechanical response.

Data-driven methods use regression, artificial neural networks or other machine-learning models to map EMG and, in some studies, kinematics or force to stiffness or to an intermediate angle/torque variable [13,14,16,50,51,52,53,54]. However, predicting motion, torque or intent does not necessarily constitute stiffness estimation unless the subsequent mapping to stiffness is explicitly defined and validated. This distinction is particularly relevant to the studies by Li et al. [14] and Zhao et al. [54], in which stiffness is incorporated into impedance-control or movement-estimation models, while experimental validation primarily focuses on task performance or motion rather than agreement with an independent joint-stiffness reference. Consequently, their results provide evidence of the performance of the proposed frameworks without directly establishing stiffness-estimation accuracy.

Beyond these validation considerations, data-driven approaches may require substantial computational resources during training, although inference is often relatively fast. Their reliability also depends on the representativeness of the training data, with potential limitations arising from small datasets, data leakage between training and test sets, and generalization to subjects, postures, loads or electrode placements not represented during training.

Model-based inference propagates neural activation through Hill/Voigt or neuromusculoskeletal dynamics and joint geometry [55,56,57,58]. Physiological interpretability is gained at the cost of parameter identifiability and calibration. EMG requirements should be stated technically: electrode location and orientation, crosstalk control, amplitude normalization, filtering and electromechanical delay, activation dynamics, signal-quality rejection and cross-session recalibration all affect the estimated muscle force and stiffness. Tendon compliance, moment arms, optimal fiber length and activation-dependent short-range stiffness are additional model assumptions.

Validation may use RMSE, normalized RMSE, coefficient of determination, variance accounted for, repeatability or agreement with an instrumented perturbation estimate. Table 4 summarizes the non-perturbative studies and summarizes their reported validation procedures and outcomes.

**Table 4:** Summary of non-perturbative joint and endpoint stiffness inference methods. M.M., M.L., ANN, SVR, D–P, F/E, RUD, DoF and NR denote musculoskeletal model, machine learning, artificial neural network, support vector regression, dorsiflexion–plantarflexion, flexion–extension, radio-ulnar deviation, degree(s) of freedom and not reported, respectively. *N* denotes the number of participants (DB: database-based study). A number without a population descriptor refers to able-bodied subjects. Reported outcomes describe different validation targets; torque and angle errors, classification accuracy, model-target correlations and computation time are not direct measures of stiffness-estimation accuracy. K-ref. indicates direct comparison of estimated stiffness with an independent reference or known value (✓, reported; and ×, not reported).

Ref.	Method	Joints	DoF	N	Validation	K Ref.	Outcomes
[50]	M.L. (ANN) + M.M.	Elbow	1: F/E	9	Perturbation-based reference	✓	Stiffness RMSE: 8.68 Nm/rad
[51]	M.L. (SVR) + M.M.	Knee	1: F/E	5	10-fold CV; instrumented comparison	×	SVM classification: 96.7%
[52]	M.L. (ANN)	Shoulder, elbow	2: planar	1	Independent trials vs. model targets	×	Stiffness correlation: R>0.9
[55]	M.M. (myokinetic)	Shoulder, elbow	2: planar	9	Perturbation-based reference	✓	NR
[59]	M.M. (OpenSim)	Upper limb	7	8	Comparison of task modalities	×	Stiffness difference: p<0.05
[57]	M.M. (OpenSim)	Ankle	1: D–P	5	Perturbation-based reference	✓	Stiffness RMSE: 0.77 Nm/rad (median)
[58]	M.M. (OpenSim)	Ankle	1: D–P	12	Joint, muscle and tendon references	✓	Mean nRMSE: ∼20%
[60]	M.M. (OpenSim)	Knee, ankle	2: F/E, D–P	5	Experimental joint-moment fit	×	NR
[61]	M.M. (OpenSim)	Wrist	2: F/E, RUD	DB	Perturbation reference; sensitivity	✓	Stiffness increase: 113% (model effect)
[62]	M.M. (sEMG-driven)	Knee	1: F/E	3	Torque fit; assistance experiments	×	Torque nRMSE: <7.21%
[63]	M.M. (Hill)	Elbow	1: F/E	5	Motion reconstruction across loads	×	Angle RMSE: 0.19–0.25 rad
[64]	M.M. (Hill) + optimization	Elbow	1: F/E	10	Independent tempo/load tasks	×	Angle deviation: ≤8.1% ROM
[56]	M.M. + cubature filter	Ankle	1: D–P	5	Simulation; test-bench torque	×	Torque RMS error: 1.27 Nm
[3]	M.M. + Kalman filter	Knee	1: F/E	5	Simulation; test-bench torque	×	Filter time: 6.29±1.77 ms
[65]	M.M. (EMG-guided)	Knee	1: F/E	5	Perturbation-based reference	✓	Stiffness error: ∼21% (high co-contraction)
[66]	M.M. (Hill)	Hip, knee	2/leg: F/E	5	Torque fit; gait-control comparison	×	Torque *R*: 0.92 (hip), 0.94 (knee)
[13]	EMG–kinematic mapping	Endpoint	2-D arm	NR	Tele-impedance tasks	×	NR
[16]	EMG-based impedance	Upper arm	1: F/E	8	EMG-model fit; hand control	×	Model $R^{2}>0.99$
[53]	LQE; force–stiffness model	Endpoint	Planar	NR	HRI/teleoperation tests	×	NR
[14]	EMG-based variable impedance	Upper limb	NR	NR	Teleoperation comparison	×	NR
[54]	M.M. + state-space ANN	Wrist/hand	Multi-DoF	NR	Cross-load motion tests	×	NR

Shen et al. [50] illustrate a hybrid data-driven and analytical pipeline. An improved ant-colony-optimization generalized-regression neural network estimated sagittal-plane elbow angle from biceps and triceps EMG. The predicted angle entered an analytical torque model,

(11) $$\tau =\sum_{i=1}^{n}\left(\frac{C_{1i}\theta E_{1i}}{\lambda (\theta )}\right)-\left(\frac{C_{2i}E_{2i}}{\lambda (\theta )}\right)+C_{3}\theta +\left(C_{4}\theta +C_{5}\right)\frac{\mu (\theta )}{\lambda (\theta )}$$

and a calibrated stiffness–torque mapping index (STMI) was converted to stiffness by

(12) $$K_{q}=D_{1}\cdot STMI+D_{2},$$

where $D_{1}$ and $D_{2}$ are calibrated coefficients. The setup combined two EMG channels with tension and angular sensing. The reported stiffness-estimation RMSE was 8.68±0.008Nm/rad and $R^{2}=0.9109\pm 0.0002$.

EMG-driven models may represent all muscles crossing the joint [57,58,59,60,61,62] or a reduced set of dominant agonist–antagonist muscles [3,56,63,64,65,66]. Both require subject-specific or population-based parameter calibration because muscle–tendon geometry, strength and activation dynamics vary across individuals. Cop et al. [57] compared an ankle OpenSim model calibrated to torque alone with one calibrated jointly to torque and an instrumented perturbation-based stiffness estimate. A dynamometer, encoder, torque transducer, footplate and EMG system provided the reference signals (Figure 10).

For *N* muscles crossing the joint, net joint stiffness was expressed as

(13) $$K_{q}=\sum_{i=1}^{N}\left(K_{i}^{m}l_{i}^{2}-\frac{\partial l_{i}}{\partial \theta }F_{i}^{m}\right),$$

where θ is joint angle, $K_{i}^{m}=\partial F_{i}^{m}/\partial l_{m_{i}}$ is local muscle stiffness, $l_{m_{i}}$ is muscle length, $l_{i}$ is the muscle moment arm and $F_{i}^{m}$ is muscle force. EMG enters Equation (13) through the preceding activation dynamics: the processed EMG envelope drives muscle activation, activation and musculotendon state determine $F_{i}^{m}$ and the derivative of that force–length relation determines $K_{i}^{m}$. Calibration to both torque and the perturbation-based stiffness estimate reduced median normalized RMSE by 0.32 and increased median $R^{2}$ by 0.38 relative to torque-only calibration; the resulting median RMSE and $R^{2}$ were 0.77Nm/rad and 0.67.

Computational feasibility depends on model size, solver, calibration schedule and hardware; full models are not intrinsically incompatible with real-time use. Reduced models trade anatomical completeness for fewer parameters and lower computation. Radmilović et al. [64] represented the elbow with biceps and triceps during a modified reach-and-retrieve task, used EMG and IMU measurements, calibrated Hill parameters with a genetic algorithm and estimated torque–angle stiffness recursively. The maximum angle-fit deviation was 8.1% of the elbow range; this is a calibration-fit outcome rather than stiffness error. Estimated stiffness spanned 1.75–7.00Nm/rad and 2.20–5.25Nm/rad in the two tested conditions.

At class level, non-perturbative inference offers continuous operation without deliberate task disturbance. Model-based approaches provide interpretable intermediate quantities, whereas data-driven approaches can approximate nonlinear mappings with low inference latency. Both require validation outside calibration conditions. Appropriate evidence includes cross-session and cross-subject testing, sensitivity to electrode shift and load/posture changes.

## 4. Discussion

Human joint and endpoint stiffness describe the local elastic behavior of the neuromusculoskeletal system in different coordinate spaces. The reviewed literature uses two primary sources of identification information—mechanical perturbation response and non-perturbative inference—implemented with different devices, sensors and computational models. The principal comparative finding is not that one implementation is most accurate, but that reported outcomes answer different validation questions under different postures, tasks, populations and reference procedures.

### 4.1. Critical Comparison and Benchmarking Framework

The absence of a standardized benchmark is a major obstacle to quantitative comparison. RMSE and normalized RMSE quantify error against a selected reference; $R^{2}$ and variance account for describe model fit rather than agreement; group differences indicate discriminative validity; and test–retest statistics assess repeatability. An instrumented perturbation estimate provides a useful comparator, although it cannot be considered an absolute ground truth as it depends on actuator dynamics, calibration, alignment, perturbation characteristics, operating conditions, and model assumptions.

Importantly, the role of mechanical perturbations differs between estimation approaches. In perturbation-response identification, perturbations provide the mechanical input from which stiffness is directly estimated. In non-perturbative inference, perturbation-based measurements may instead serve as calibration targets, independent validation references, or both. Therefore, agreement on calibration data should be distinguished from validation on independent test data, while repeatability and external generalization provide complementary evidence of estimator reliability.

Table 5, Table 6 and Table 7 combine evidence reported in the reviewed studies with qualitative, application-oriented assessments proposed by the authors. Validation evidence is summarized using named categories: no quantitative validation reported; plausibility, known-condition effects or group discrimination; agreement with an instrumented comparator; repeatability or test–retest evidence; and generalization to independent conditions, sessions or participants. These categories are nonexclusive: a study may contribute to more than one category. They represent complementary forms of evidence and are not combined into a cumulative accuracy score or interpreted as an ordinal ranking. Agreement with a comparator is distinguished according to whether the data were used for calibration or reserved for independent validation. Model-fit and simulation-recovery outcomes are identified as such and do not by themselves establish independent agreement.

These evidence categories can be illustrated through representative studies. Joghtaei et al. [23] reported differences in passive knee stiffness between clinical groups, providing known-condition and group-discrimination evidence. Cop et al. [57] compared model-based ankle stiffness estimates with perturbation-derived references using data not employed for calibration, providing agreement evidence on independent data. Formica et al. [21] repeated wrist stiffness measurements across two sessions separated by one month, providing repeatability evidence. Generalization evidence requires validation on independent participants, sessions or external datasets, rather than additional trials from the same subject-specific calibration protocol.

**Table 5:** Validation evidence in the application-oriented benchmarking framework. The qualitative assessments are informed by representative studies and do not constitute a ranking of estimation accuracy.

Implementation Approach	Validation Evidence	Primary Class
Manual or clinician-applied perturbation [22,23]	Plausibility or group-discrimination evidence; quantitative stiffness validation may be absent	Perturbation response
Robot or exoskeleton perturbation [21,45]	Instrumented-comparator and repeatability evidence, where reported; comparator is not absolute ground truth	Perturbation response
Mechatronic orthosis or joint-specific platform [30,31]	Comparator agreement, model fit or test–retest evidence, as reported	Perturbation response
Dynamic system-ID approaches [19,39]	Simulation recovery and experimental model fit; independent generalization requires separate testing	Perturbation response
Machine-learning and deep-learning approaches [50,51]	Calibration and held-out testing; external generalization requires independent subjects or datasets	Non-perturbative inference
EMG-driven musculoskeletal or Hill-type models [57,58]	Model calibration and agreement with experimental references; repeatability and generalization depend on validation design	Non-perturbative inference
Computational model-based inferences [3,61]	Simulation-based validation and/or agreement with experimental references; independent validation depends on study design	Non-perturbative inference

**Table 6:** Author-proposed assessment of online suitability and robustness considerations, informed by the studies summarized in Table 5. Measured real-time estimation latency and the computing hardware used were not reported in the cited studies. Online-use descriptions are, therefore, qualitative and implementation-dependent, not standardized performance ratings; feasibility depends on the algorithm, data window, calibration, acquisition pipeline and computing hardware.

Implementation Approach	Online Use	Robustness
Manual or clinician-applied perturbation	Not designed for continuous online use	Operator-dependent force direction, amplitude and velocity; limited repeatability
Robot or exoskeleton perturbation	Suitable for assessment; limited for control if perturbations disturb the task	Robot dynamics, alignment, gravity compensation, perturbation bandwidth, safety constraints
Mechatronic orthosis or joint-specific platform	Possible for repeated assessments; device-specific for online use	Joint-specific geometry, orthosis fit, friction, range of motion, calibration
Dynamic system-ID approaches	Potentially feasible when algorithms are optimized and data windows are short; implementation-dependent	Time-varying behavior, non-stationarity, input richness, reflex contributions
Machine-learning and deep-learning approaches	Potentially feasible after training; depends on model size and acquisition pipeline	Dataset shift, population imbalance, limited interpretability
EMG-driven musculoskeletal or Hill-type models	Potentially feasible after calibration, especially with reduced models; depends on solver and hardware	Subject-specific parameters, EMG normalization, tendon compliance, moment arms, co-contraction
Computational model-based inferences	Implementation-dependent; often offline unless the model is simplified	Model assumptions, parameter identifiability, lack of direct validation

**Table 7:** Potential applications and author-proposed reporting recommendations for the implementation approaches in Table 5.

Implementation Approach	Application	Reporting
Manual or clinician-applied perturbation	Clinical screening; low-resource assessment	Applied force, direction, speed, joint posture, examiner training, number of repetitions
Robot or exoskeleton perturbation	Rehabilitation assessment; impedance identification; controlled experiments	Actuator type, control mode, perturbation amplitude/frequency, alignment, inertia/damping compensation
Mechatronic orthosis or joint-specific platform	Ankle, knee, elbow and wrist assessment	Calibration, friction compensation, joint-axis alignment, sampling rate, filtering, inertia assumptions
Dynamic system-ID approaches	Time-varying impedance; gait; reaching; postural control	Input/output signals, estimation window, perturbation spectrum, VAF, bias, variance, rise time
Machine-learning and deep-learning approaches	Robot control; intent recognition; online estimation	Dataset size, split strategy, cross-subject validation, inference time, preprocessing, normalization, code/data availability
EMG-driven musculoskeletal or Hill-type models	Physiological stiffness estimation; rehabilitation robotics; prosthetic impedance control	EMG placement, MVC normalization, filters, muscle set, calibration objective, model parameters, computation time
Computational model-based inferences	Hypothesis testing; model comparison; protocol design	Model equations, parameters, sensitivity analysis, validation dataset, assumptions

### 4.2. Accuracy and Validation Across Methods

Perturbation-response systems offer experimental control and a physically explicit input–output relation, but task disturbance, device compensation and local linearity constrain ecological use. Non-perturbative inference avoids a deliberate online perturbation and may support continuous control; its validity depends on calibration, signal stability, parameter identifiability and generalization. EMG-driven models provide interpretable muscle-level intermediates, whereas machine learning can provide low-latency nonlinear inference. Neither can be considered more accurate without head-to-head testing on the same quantity, coordinate space, posture, task and reference protocol.

Beyond these methodological considerations, clinical applicability also requires reproducible measurements across operators and sessions. In this context, differences in operator training and the execution of manual perturbations, as well as variations in device fitting and alignment with anatomical joint axes, may introduce additional variability in stiffness estimates. These factors highlight the importance of standardized assessment protocols, documented alignment and calibration procedures, and repeated-measure evaluations to establish the consistency of stiffness estimates across clinical settings.

Future studies should report estimation accuracy, repeatability, uncertainty and computational performance. Perturbation-response methods should be evaluated across different perturbation conditions and joint postures, whereas non-perturbative approaches should be validated across sessions, participants and operating conditions. The importance of accounting for posture and stabilizing function is further supported by [67], who showed that experimentally restricting the wrist’s ability to generate stabilizing torque substantially reduced the maximum pushing force across two arm configurations. Although the study did not directly estimate a stiffness matrix, its findings highlight how coordinate definitions, limb posture and the stabilizing role of endpoint stiffness can critically affect the interpretation and validation of stiffness-estimation methods.

For time-varying estimates, global fit should be complemented by rise time, bias and random error during stiffness transitions [19].

### 4.3. Application-Oriented Method Selection

To support the selection of the most appropriate stiffness-estimation method for a specific application, an application-oriented flowchart is provided in Figure 11. The flowchart is not intended to rank methods according to absolute accuracy, but rather to guide the reader through a sequence of practical questions related to the target stiffness construct, experimental constraints and available sensing or modeling resources.

Once the target quantity and its biomechanical context have been defined, the primary question is whether a known mechanical identification input can be applied during the target task. If so, perturbation-response identification is selected and the participant’s operating state determines the appropriate protocol: passive assessment under relaxed conditions, quasi-static identification during active-static tasks or time-varying identification during active movement. Conversely, when an identification input cannot be applied, stiffness must be inferred non-perturbatively. The choice then depends on the validated information available: calibrated physiological measurements combined with a physics-based relationship support model-based estimation, whereas labeled reference stiffness data enable data-driven estimation. If neither is available, reference data must first be collected to calibrate or train and validate the estimator.

The practical application of the decision tree can be illustrated through two scenarios involving clinical assessment and continuous robotic control. Consider, for instance, the assessment of passive knee joint stiffness under relaxed conditions. The target quantity is the rotational stiffness of the knee, expressed in joint coordinates. Since the assessment does not involve voluntary movement, controlled mechanical perturbations can be applied to characterize the joint response. Following the decision tree, this leads to perturbation-response identification using a passive protocol. A representative implementation is the pendulum test, in which knee stiffness is estimated from the oscillatory response of the lower leg following release. Once the method has been selected, its feasibility depends on whether the recorded response allows the relevant mechanical parameters to be identified, while accounting for gravitational and damping contributions. The applied movement must also be acceptable for the participant, and the measurements should provide sufficient repeatability for the intended assessment.

A different methodological choice arises when continuous elbow-stiffness estimation is required for controlling a robotic prosthesis. In this case, the target is the time-varying rotational stiffness of the elbow during voluntary movement. Applying repeated mechanical perturbations would interfere with the intended task, making perturbation-response identification unsuitable for continuous operation. The decision tree, therefore, leads to non-perturbative inference. When calibrated EMG measurements, joint kinematics and an appropriate neuromusculoskeletal model are available, a model-based approach can be selected to estimate stiffness from muscle activation and the current biomechanical state. Mechanical perturbations may still be employed during calibration or validation, but are not required during online estimation. Following method selection, parameter identifiability, estimation reliability across relevant movements and loading conditions, and acquisition and computation latency must be evaluated to determine whether the approach meets the control requirements.

These examples illustrate how the proposed decision tree guides the selection of an appropriate stiffness-estimation approach according to the target quantity and experimental constraints, while the subsequent assessment of parameter identifiability, acceptable disturbance, and computational latency determines its practical feasibility.

## 5. Conclusions and Future Perspectives

This review synthesized methods for estimating human joint and endpoint stiffness, emphasizing the distinction between coordinate spaces and between stiffness and other components of mechanical impedance. Perturbation-response methods provide an explicit local mechanical relationship and are particularly suited to controlled assessment when mechanical excitation is acceptable. Conversely, non-perturbative model-based and data-driven methods are more suitable for continuous estimation when imposed perturbations would interfere with the task, although their reliability depends on calibration and validation beyond the conditions used for model development. Neither approach is universally superior, and perturbation-based estimates should not be regarded as absolute ground truth without considering model and device uncertainty. Furthermore, several studies involved small cohorts, predominantly comprising healthy participants, which limits the generalizability of the available evidence and calls for caution when extending the proposed recommendations to clinical applications.

Addressing these limitations requires construct-specific reference protocols and shared datasets that enable methods to be evaluated under comparable biomechanical and task conditions. Such efforts should promote more consistent comparisons of stiffness estimates across methods, considering differences in joint configuration, muscle activation and experimental protocols, while assessing both estimation accuracy and measurement repeatability. At the same time, extending the validation of non-perturbative approaches beyond calibration conditions, including different movements, loading conditions and experimental sessions, would provide further insight into their reliability and generalizability. In applications requiring continuous stiffness estimation, these evaluations should also consider computational latency and agreement with independent experimental references to support their translation into real-time robotic systems.

## Figures and Tables

**Figure 1:**
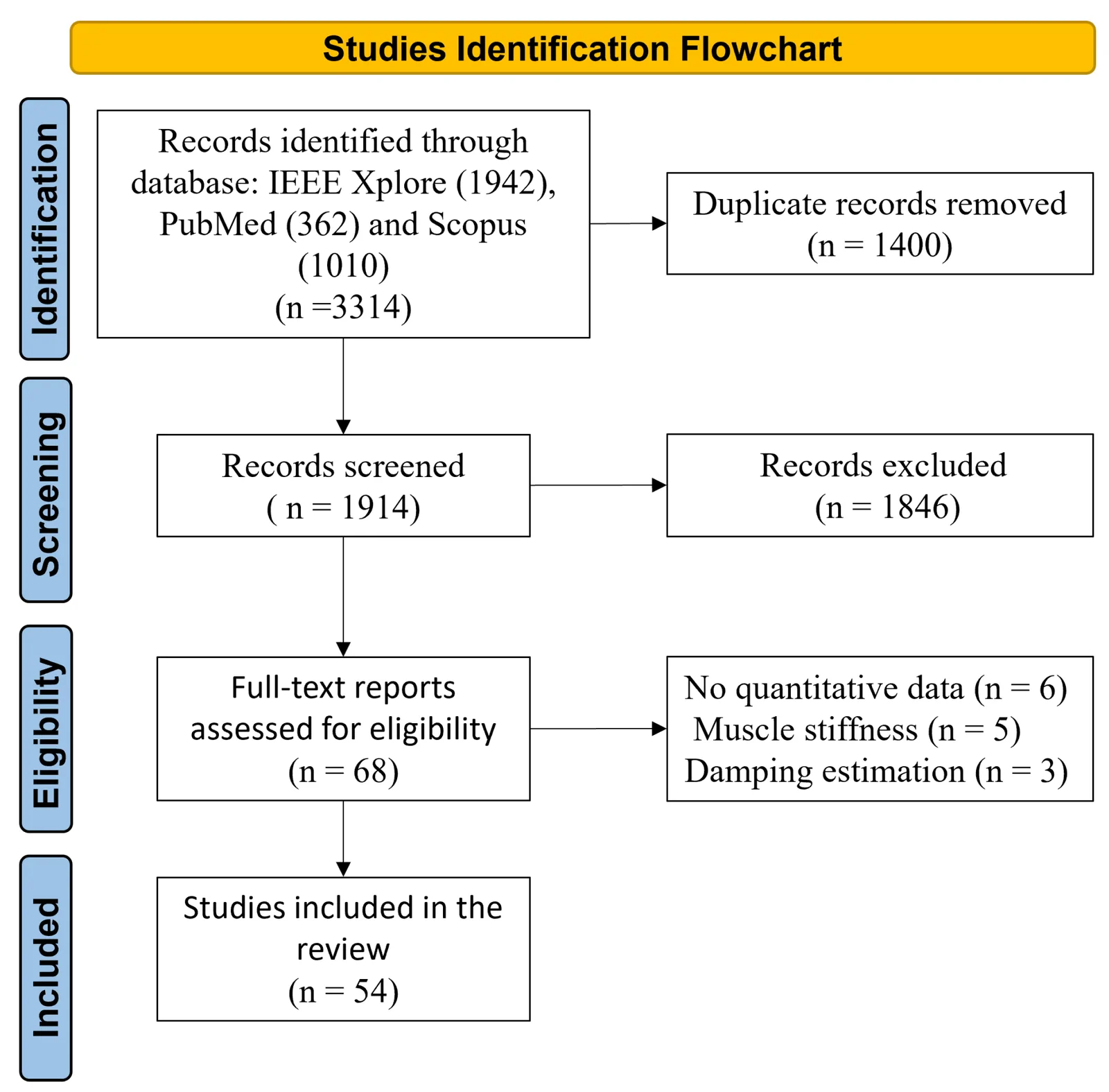
Literature-selection flow diagram reporting the identification, screening, eligibility and inclusion of studies.

**Figure 2:**
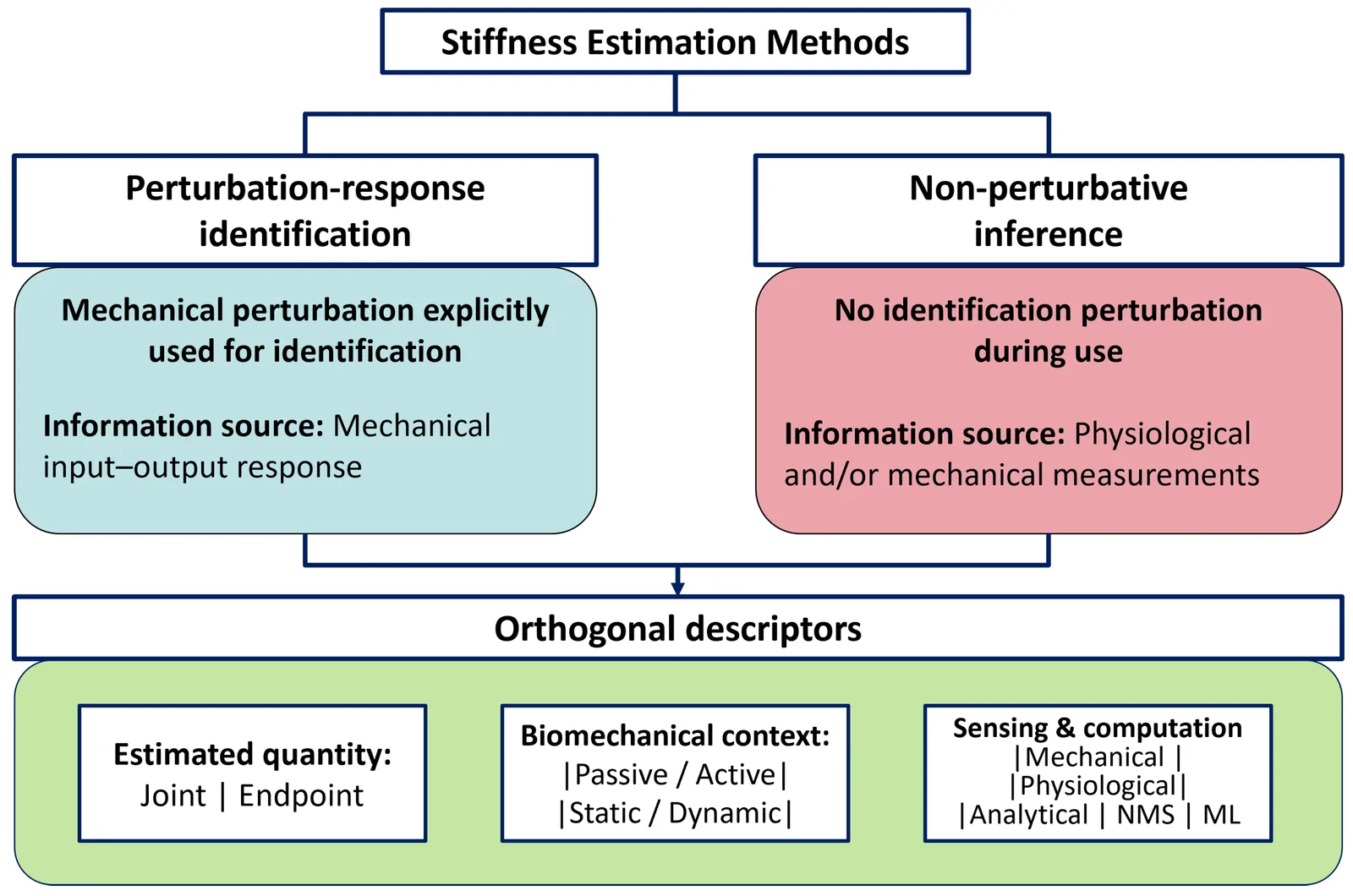
Taxonomy of human joint and endpoint stiffness-estimation methods.

**Figure 3:**
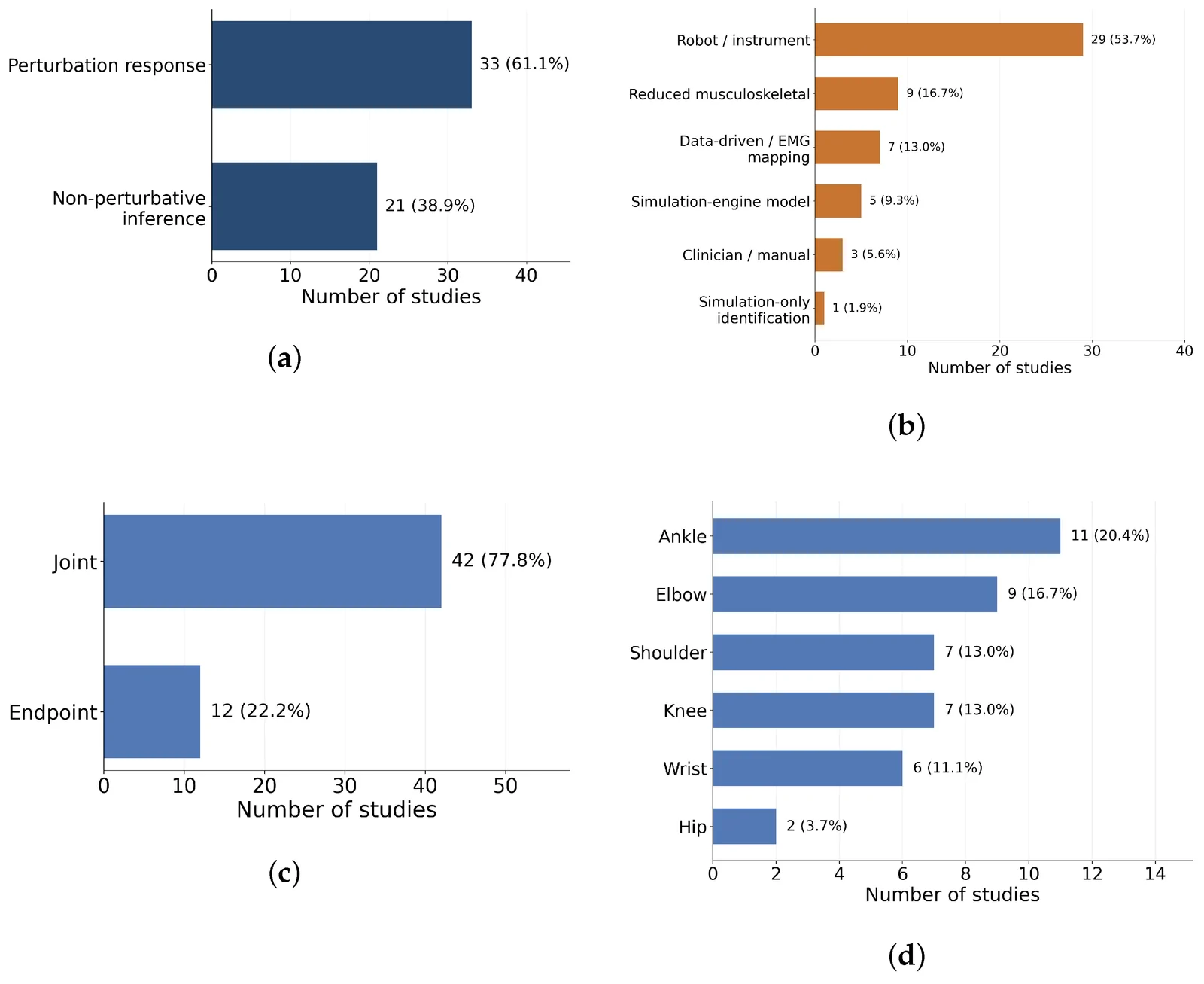
Distribution of the 54 studies in the revised synthesis according to primary identification principle (**a**), dominant implementation technology (**b**), primary stiffness coordinate space (**c**), and primary anatomical joint for joint-space studies (**d**). Counts and percentages are shown.

**Figure 4:**
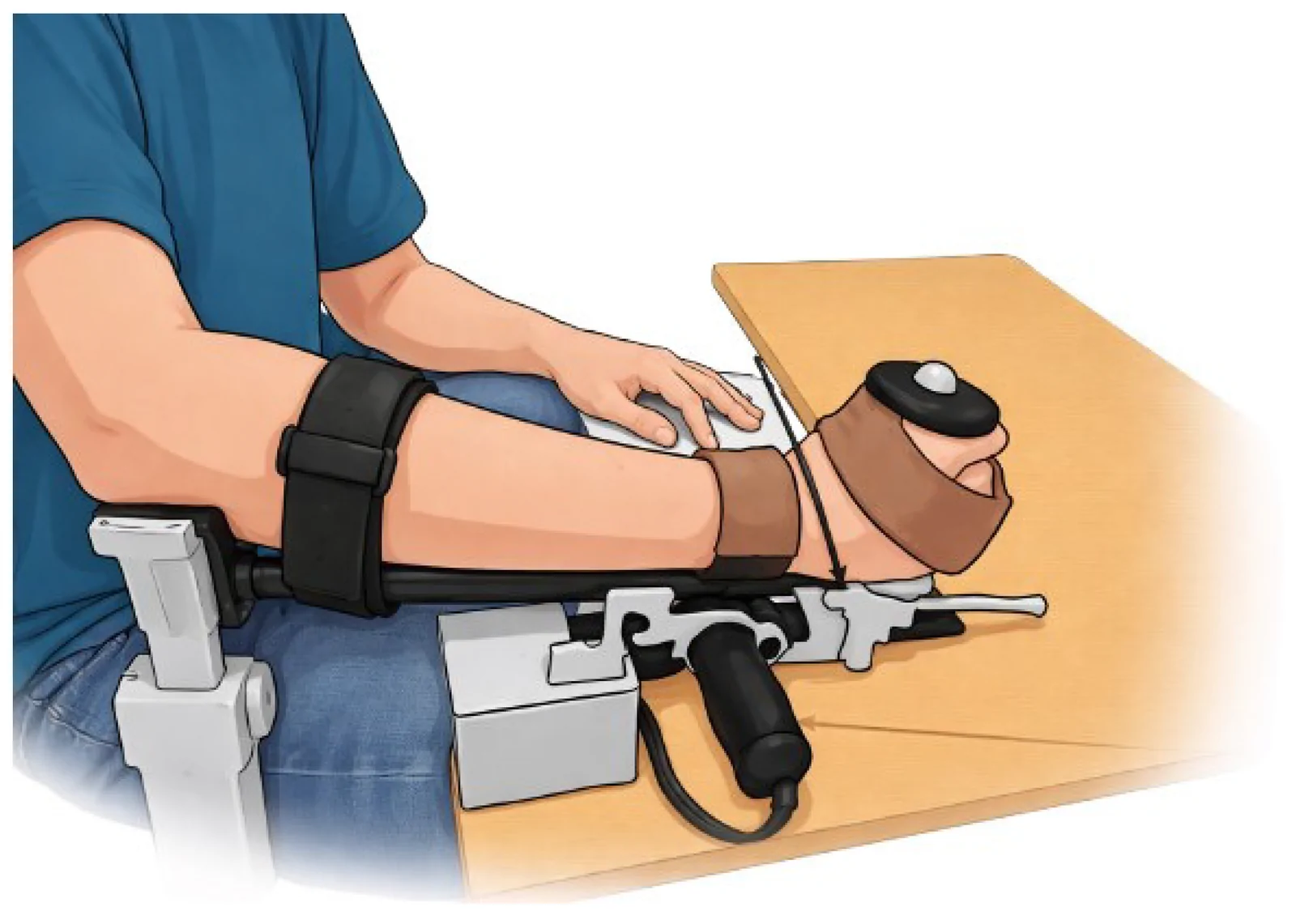
Example of the experimental setup used in [21].

**Figure 5:**
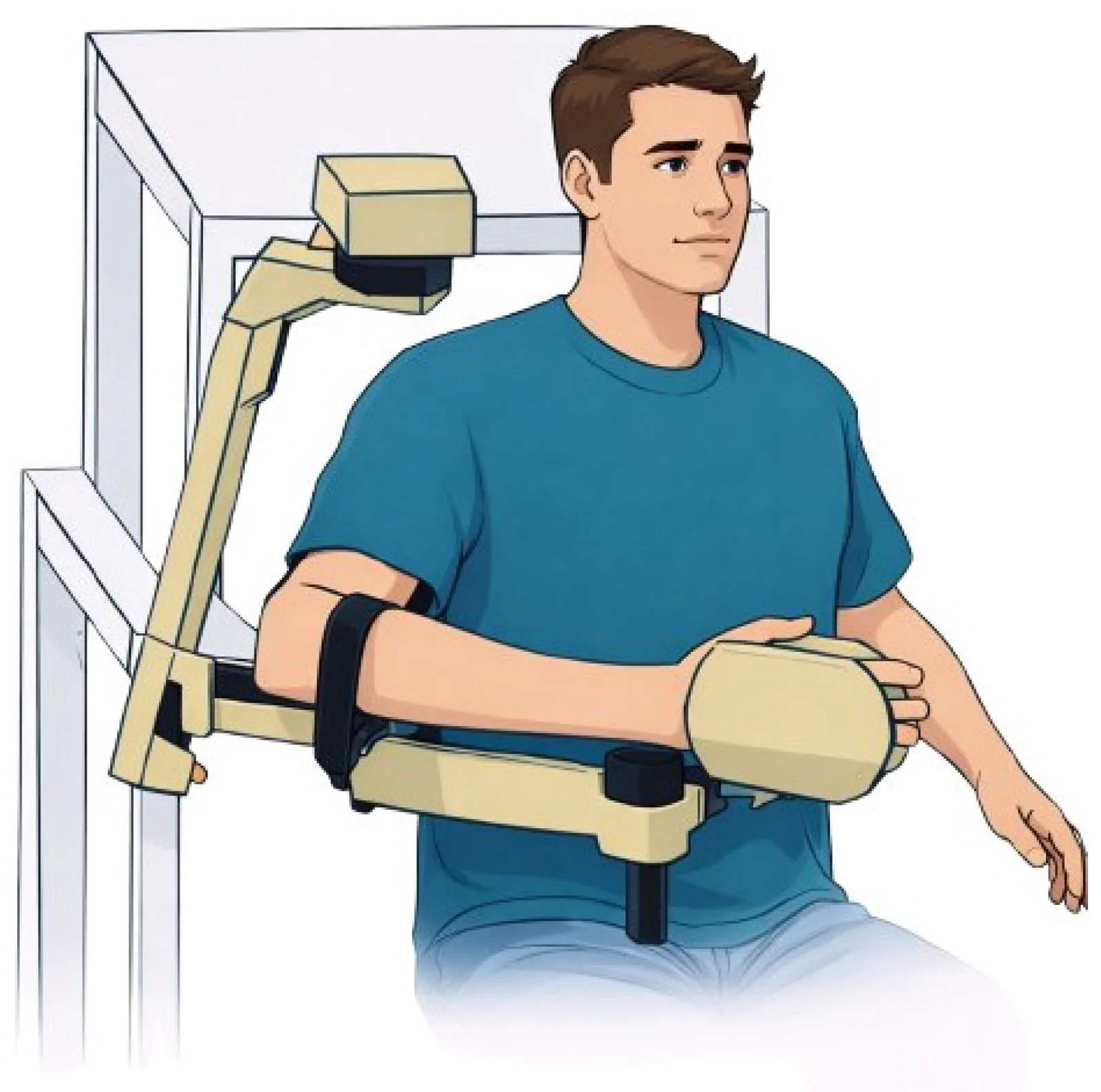
Example of the experimental setup used in [12].

**Figure 6:**
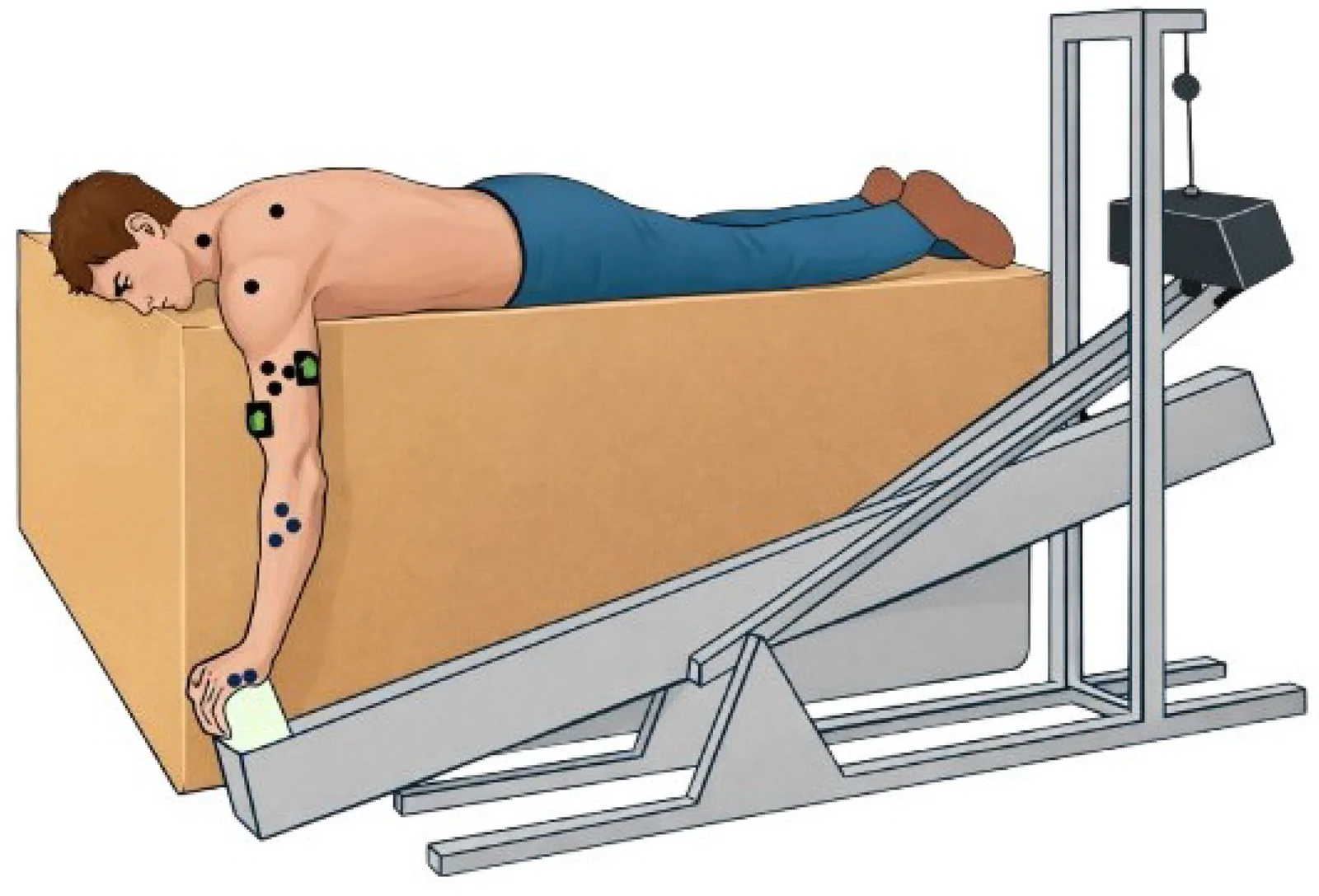
Example of the experimental setup used in [29].

**Figure 7:**
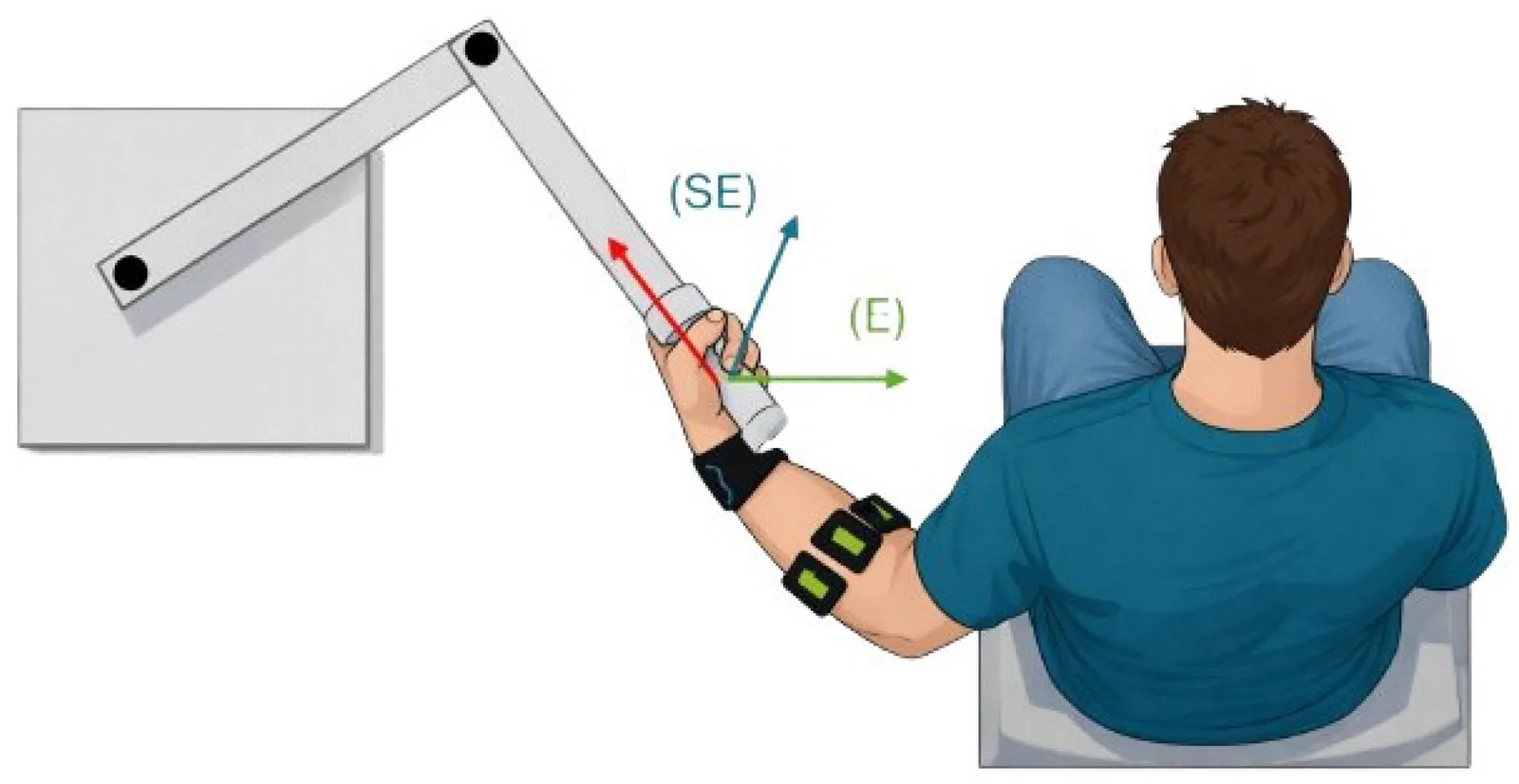
Example of the experimental setup used in [32].

**Figure 9:**
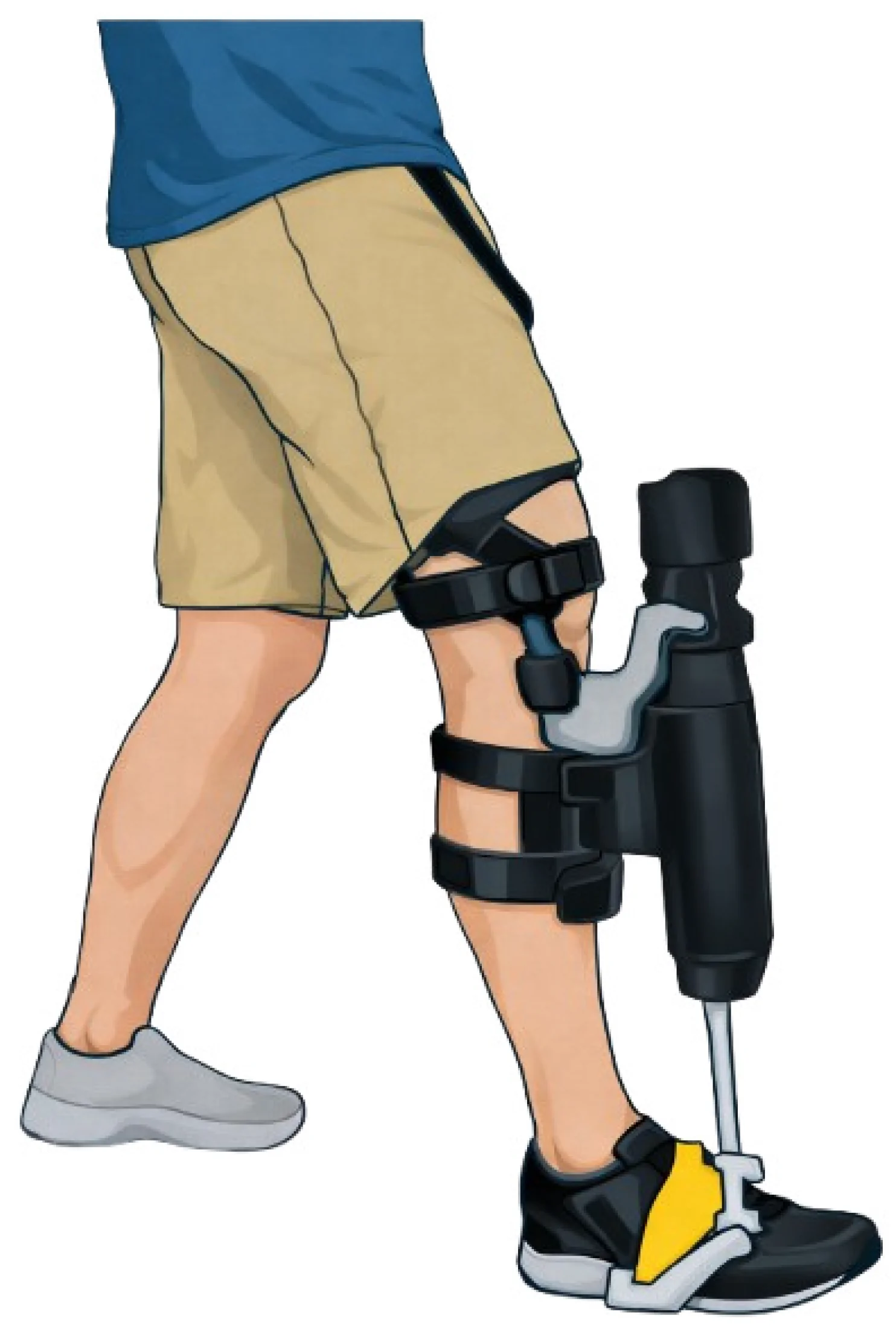
Example of the experimental setup used in [45].

**Figure 10:**
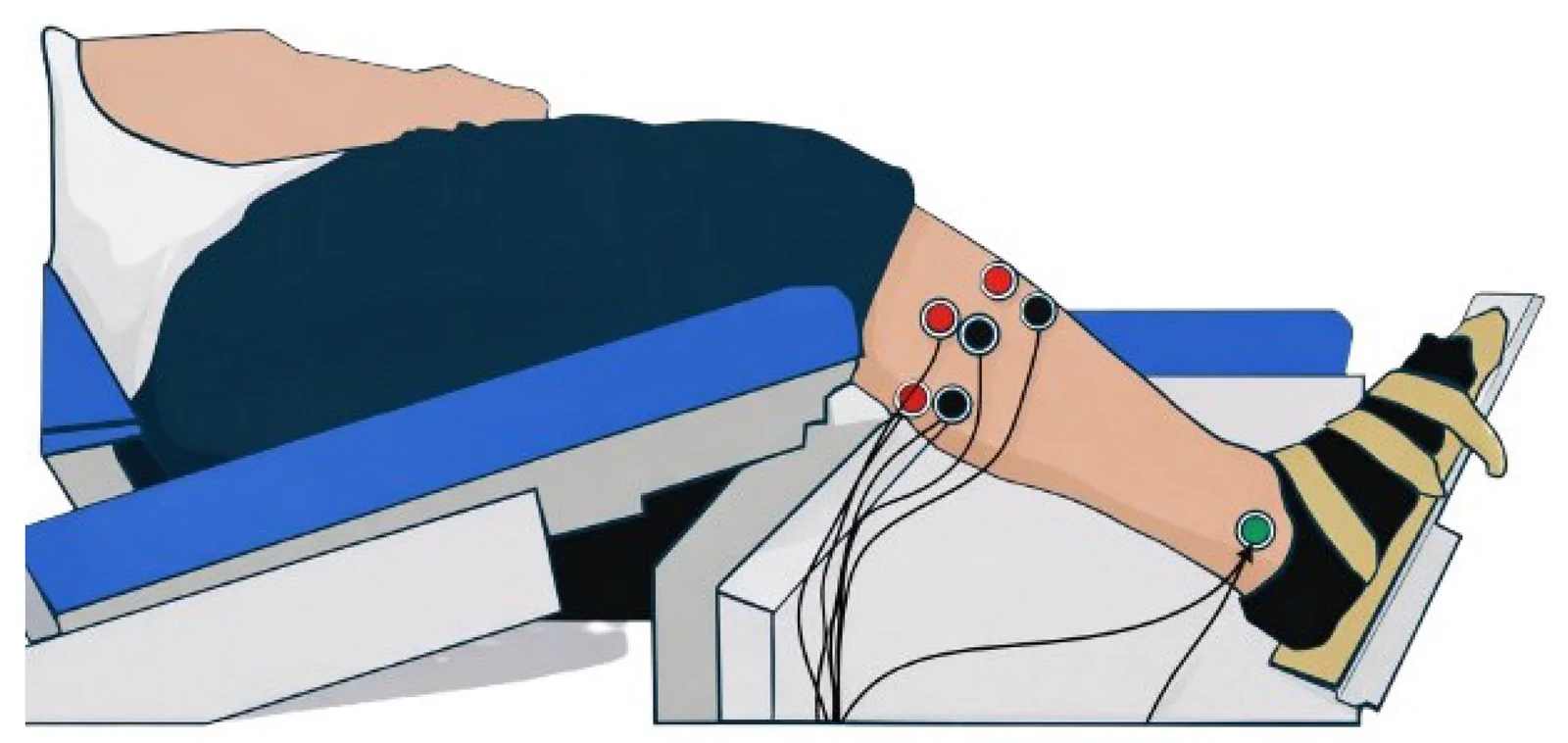
Example of the experimental setup used in [57].

**Figure 11:**
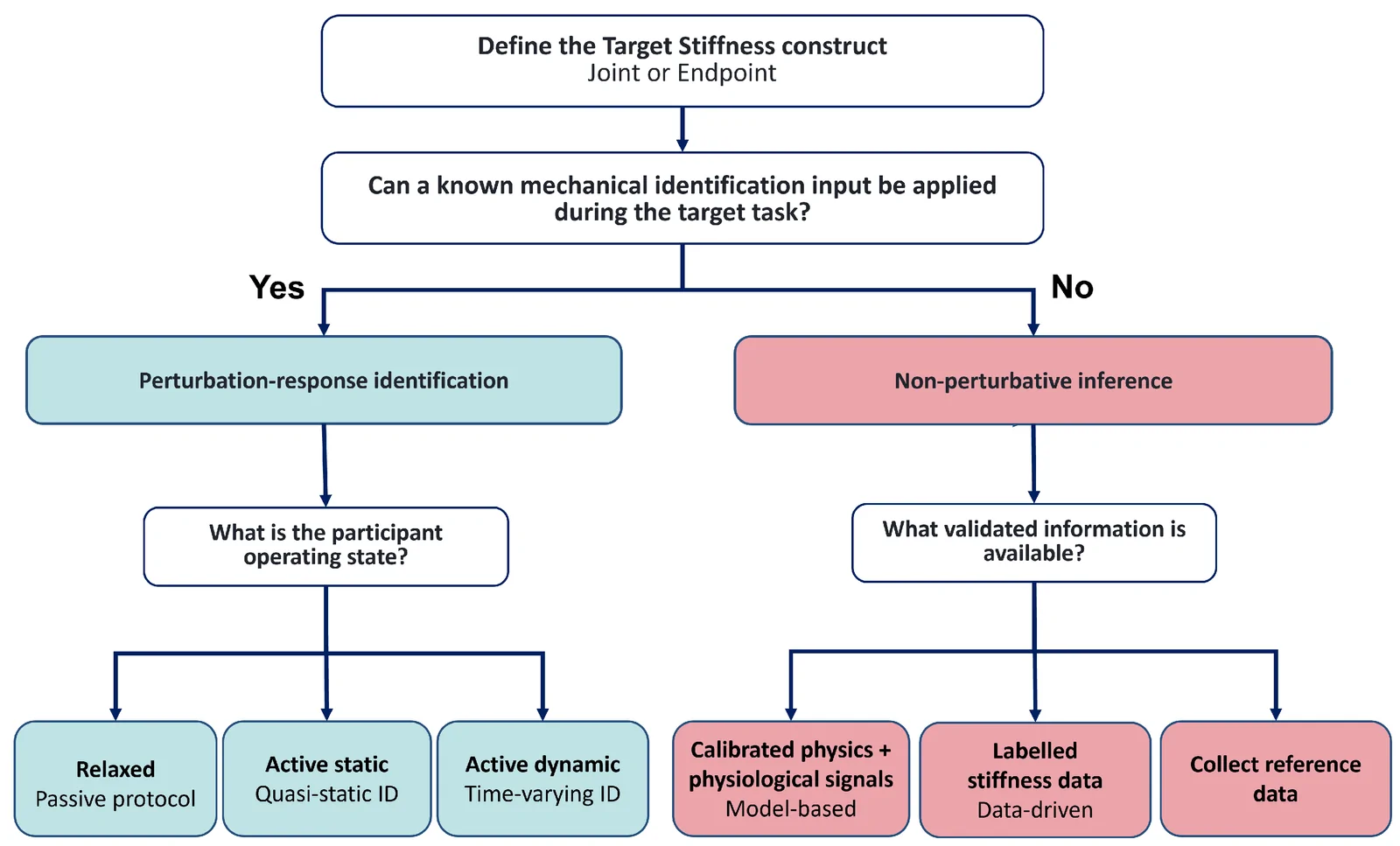
Application-oriented decision tree for joint- and endpoint-stiffness estimation. Method selection considers the target stiffness construct, the availability of a mechanical identification input, the operating state, and the available validated information.

## Data Availability

No new data were created or analyzed in this review. The paper synthesizes information from previously published peer-reviewed studies.
